# Transcriptomic evidence of lung repair in paediatric ARDS survival

**DOI:** 10.1002/ctm2.1366

**Published:** 2023-08-17

**Authors:** Licheng Song, Kuan Li, Xiaoyang Hong, Kun Xiao, Guoxin Mo, Mengli Zheng, Fei Xie, Yuhong Liu, Pengfei Liu, Tianyu Sun, Bo Wang, Qiushuang Feng, Aiguo Zhou, Chen Yao, Jing Wang, Huaiyong Chen, Lixin Xie

**Affiliations:** ^1^ College of Pulmonary and Critical Care Medicine 8th Medical Center of Chinese PLA General Hospital Beijing People's Republic of China; ^2^ Department of Basic Medicine Haihe Hospital Tianjin University Tianjin People's Republic of China; ^3^ PICU Faculty of Pediatrics 7th Medical Center of Chinese PLA General Hospital Beijing People's Republic of China; ^4^ Key Research Laboratory for Infectious Disease Prevention for State Administration of Traditional Chinese Medicine Tianjin Institute of Respiratory Diseases Tianjin People's Republic of China; ^5^ Tianjin Key Laboratory of Lung Regenerative Medicine Tianjin People's Republic of China

Dear Editor,

Acute respiratory distress syndrome (ARDS) is triggered mostly by viral or bacterial infections. As the lung and immune systems are still developing in childhood, the pathophysiology of paediatric ARDS (PARDS) may differ substantially from ARDS in adults, leading to substantial differences in clinical outcomes.^1^, ^2^, ^3^ The pathological process of ARDS is divided into acute exudative, proliferative and fibrotic phases,^3^ which can be superimposed, with pro‐fibrotic pathways being triggered as early as the first day of the illness.^4^, ^5^ The efficacy of veno‐venous (VV) extracorporeal membrane oxygenation (ECMO) in patients with ARDS remains controversial^6^, ^7^ because the mortality of ARDS remains high and is closely associated with abnormal lung repair and severe complications of ECMO.^8^ Current pathological findings are based on autopsies or biopsies of non‐surviving patients with severe ARDS. Comprehensive evidence of reparative mechanisms in surviving ARDS is currently lacking. Here we present a case report of a 10‐year‐old girl who survived ARDS with an air leak under ECMO support. We performed a single‐cell RNA sequencing (scRNA‐seq) analysis of the lung biopsy sample taken from the surgical repair of the pneumothorax and tracked dynamic transcriptomic changes in peripheral blood immune cells. These preliminary data provided insight into the immune microenvironment that favours lung repair in PARDS.

**CRITICAL POINTS**

1. Monocyte‐derived macrophages and lung stromal cells in the proliferative stage of survived PARDS presented reparative phenotypes.
2. Surgical intervention promotes lung recruitment in ARDS with persistent air leakage supported by ECMO.
3. ECMO significantly improves the mitochondrial function of immune cells in PARDS.
4. Megakaryocytes and haematopoietic stem cells enhance TGF‐β communication to manage ECMO bleeding risk.

A previously healthy 10‐year‐old girl presented with fever, left chest pain and lethargy (D1; Figure 1A). Chest computed tomography (CT) showed diffuse ground‐glass opacity (GGO) in the right upper and lower lobes (Figure 1B). As dyspnoea worsened, the patient was intubated on D4. Methylprednisolone was administered systemically from D11, and the patient's body temperature gradually returned to normal (Supplementary Figure 1A). A repeat chest CT suggested diffuse progression of the GGO (Figure 1C). Neither respiratory pathogens (Supplementary Table S1) nor streptolysin O was detected. On D14, her respiratory distress worsened, and chest radiography showed severe subcutaneous emphysema and pneumomediastinum. VV ECMO was initiated on D23, and right‐sided closed thoracic drainage was performed. Post‐ECMO, a CT scan showed bilateral pneumothorax, diffuse subcutaneous emphysema, pneumomediastinum and bilateral atelectasis (Figure 1D). Moreover, next‐generation sequencing showed that the diversity and abundance of residential bacteria were sharply decreased (Supplementary Figure 1B).

**FIGURE 1:**
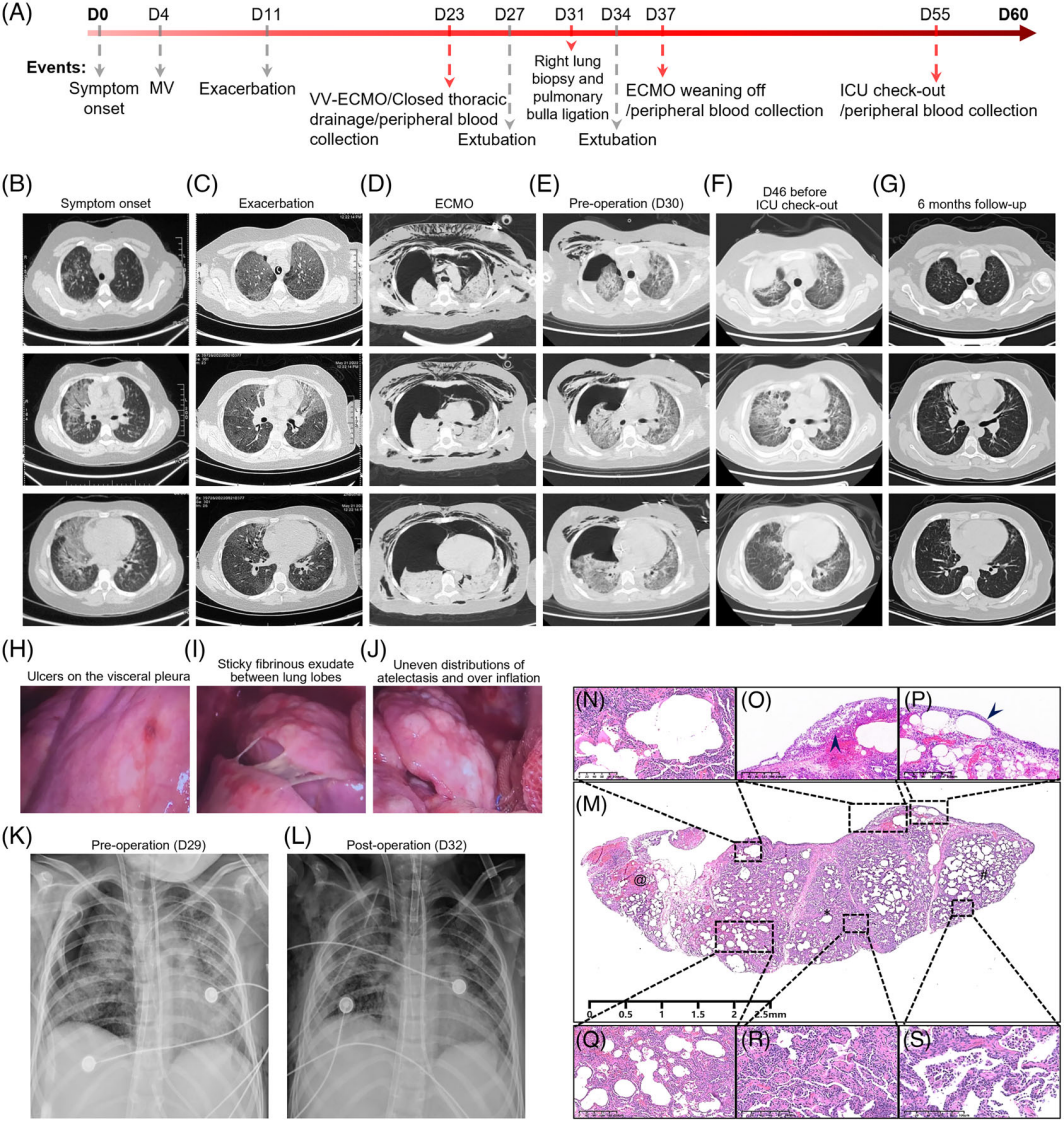
Histopathological and radiographic findings of a 10‐year‐old girl with acute respiratory distress syndrome (ARDS). (A) Clinical events are plotted or written versus time in days, with symptom onset labelled as D0 and hospital discharge labelled as D60. The dotted arrows show the major events over time. (B) Computed tomography (CT) image showing patchy ground‐glass opacity (GGO) with a crazy‐paving pattern in the right lung on the day of symptom onset. (C) Diffused GGO with superimposed septal thickening and emphysematous parenchyma are seen in the bilateral upper lobes on CT images when the disease is exacerbated. (D) Severe right pneumothorax with subcutaneous emphysema and pneumomediastinum. The mediastinum shifted to the left and compressed the left lung lobes. (E) Pre‐operative CT showing that the subcutaneous emphysema and pneumomediastinum significantly improved compared with (D), while the right pneumothorax was refractory despite placement of closed drainage in the pleural cavity for 1 week. Both lung parenchyma shows GGO and interlobular septal thickening. (F) CT performed at 9 days after extracorporeal membrane oxygenation (ECMO) weaning showing no sign of pneumothorax and complete absorption of subcutaneous emphysema and pneumomediastinum. (G) At the 6‐month follow‐up, the GGO had resolved, leaving some degree of irregular linear opacity. (H) and (I) In the video‐assisted thoracoscopic surgery (VATS) view, the right lung was stiff with ulcers on the visceral pleura (H) as well as sticky purulent exudate between the lung lobes (I). (J) During single‐lung ventilation, the lung lobes show uneven distributions of atelectasis and over‐inflation. (K) Radiograph demonstrating a right pneumothorax and leftward displacement of the heart. (L) The right lung is well inflated after ligation of the bullae. (M) Lung tissue from the right upper lobe during VATS biopsy. In the low‐magnification field, the collapsed (*) and over‐inflated (#) alveoli were irregularly distributed across the sample. Some areas showed intra‐alveolar haemorrhaging (@). (N) Some alveoli are lined by eosinophilic hyaline membranes. (O) Hyperplasia of the mesothelial cells loosely lines the pleura with high‐tension bubbles beneath the pleura (P). (Q) Some alveoli were filled with red blood cells admixed with fibrin. The collapsed alveoli were accompanied by enlarged alveoli. (R) Small fibroblastic foci were distributed throughout the collapsed alveoli. (S) Marked multifocal proliferation of type II pneumocytes, infiltration of macrophages with a broad, sometimes foamy cytoplasm and cellular debris within the alveoli and proliferation of fibrous connective tissue in the thickened alveolar septum.

With no improvement in the pneumothorax (Figure 1K), we performed video‐assisted thoracoscopic surgery with ECMO support. The right lung was tough, with some ulcers on the visceral pleura and sticky purulent exudate between the lobes (Figure 1H,I). Each lobe was unevenly re‐inflated after high‐pressuring single‐lung ventilation (Figure 1J). Two ruptured emphysematous bullae were identified on the edge of the upper lobe. A small portion of tissue from the lesion was collected for biopsy before the bullae were closed by double ligation. Haematoxylin and eosin staining of the tissue showed characteristics of the proliferative phase of ARDS (Figure 1 M–S).

The right lung re‐inflated after surgery (Figure 1L). The patient was successfully weaned off ECMO on postoperative day 6 (D37). A chest CT on D46 showed no signs of subcutaneous emphysema or pneumomediastinum (Figure 1F). A chest CT at 6 months of follow‐up showed that the lung lesions had almost completely disappeared (Figure 1G).

scRNA‐seq analysis of lung tissues suggested that genes of major histocompatibility complex (MHC) class II, MHCI and inflammation were decreased in the macrophage subsets of the ARDS subject versus controls, while genes related to lung–repair, lipid–metabolism and phagocytosis were upregulated (Supplementary Figure 2; Figure 2A–D). In the ARDS subject, extracellular matrix production and metabolism‐related genes, including CCL18, ELN and DCN, were downregulated in Early B‐Cell Factor 1 (EBF1) relatively highly expressed fibroblasts (EBF1^+^ fibroblasts) and lipofibroblasts. Genes associated with lung repair, including EGR3, TGFB1, TGFBI, AREG, FGF7, IL33, VEGFA, PDGFRA, VCAN, CTGF and TIMP1, were upregulated in the fibroblast subsets (Figure 2D; Supplementary Table S2). Furthermore, enhanced cell–cell interactions among ficolin 1 (FCN1) relatively highly expressed macrophages (FCN1^+^ macrophages), secreted phosphoprotein 1 (SPP1) relatively highly expressed macrophages (SPP1^+^ macrophages), lipofibroblasts and mesothelial cells were observed in the ARDS subject (Figure 2E). Therefore, multiple cells exhibiting a lung repair phenotype may have contributed to the survival of this child with ARDS.

**FIGURE 2:**
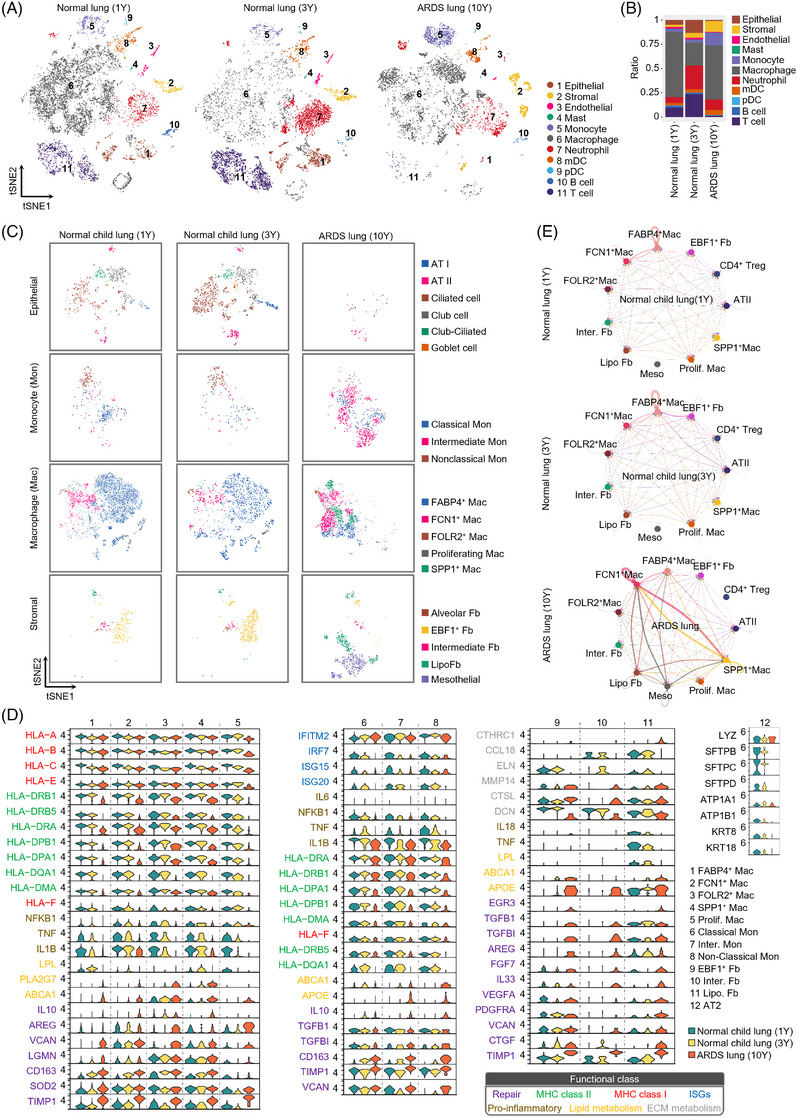
Single‐cell RNA sequencing analysis of the lung biopsy sample. (A) T‐distributed stochastic neighbour embedding (tSNE) plots of total cells of lung tissues from (left to right): 1‐year‐old boy's normal lung, 3‐year‐old boy's normal lung, and 10‐year‐old girl's acute respiratory distress syndrome (ARDS) lung. Each colour represents a cell type. The cells were pooled across all participants and are separately presented by sample. (B) Cell composition of each participant. Each cell type from the lung tissues was coloured as per the legend in panel A. (C) tSNE plots of (top to bottom): epithelial cells, monocytes, macrophages and stromal cells. (D) Violin plots of expression levels of selected genes in (left to right): macrophages, monocytes, fibroblasts and alveolar type II cells. Each colour represents a participant. Mon, monocyte; Mac, macrophage; Fb, fibroblast; Meso, mesothelial; Inter, intermediate; Prolif, proliferating (E) Cell interaction network showing interactions across alveolar type II cells, monocytes, macrophages and stromal cells (top to bottom): 1‐year‐old boy's normal lung, 3‐year‐old boy's normal lung, and 10‐year‐old girl's ARDS lung. Each colour represents a subset. All arrows indicate receptor cells. The thick lines indicate strong interactions between the ligand and receptor pairs, while the thin lines represent weak interactions. Significant interactions with *p* values < 0.05, identified using a permutation test are shown.

Next, we assayed the dynamic transcriptome of myeloid cells during ECMO (Supplementary Figure 3; Figure 3A,B). The mitochondrial and MHCII‐related genes showed an upward trend in the ARDS subject from ECMO initiation to intensive care unit (ICU) discharge (Figure 3C–F). Interferon‐stimulated gene (ISG) expression remained at low levels before ECMO weaning (Figure 3F). Interferon‐ and inflammation‐related genes were downregulated at ECMO weaning versus initiation (Figure 3F).

**FIGURE 3:**
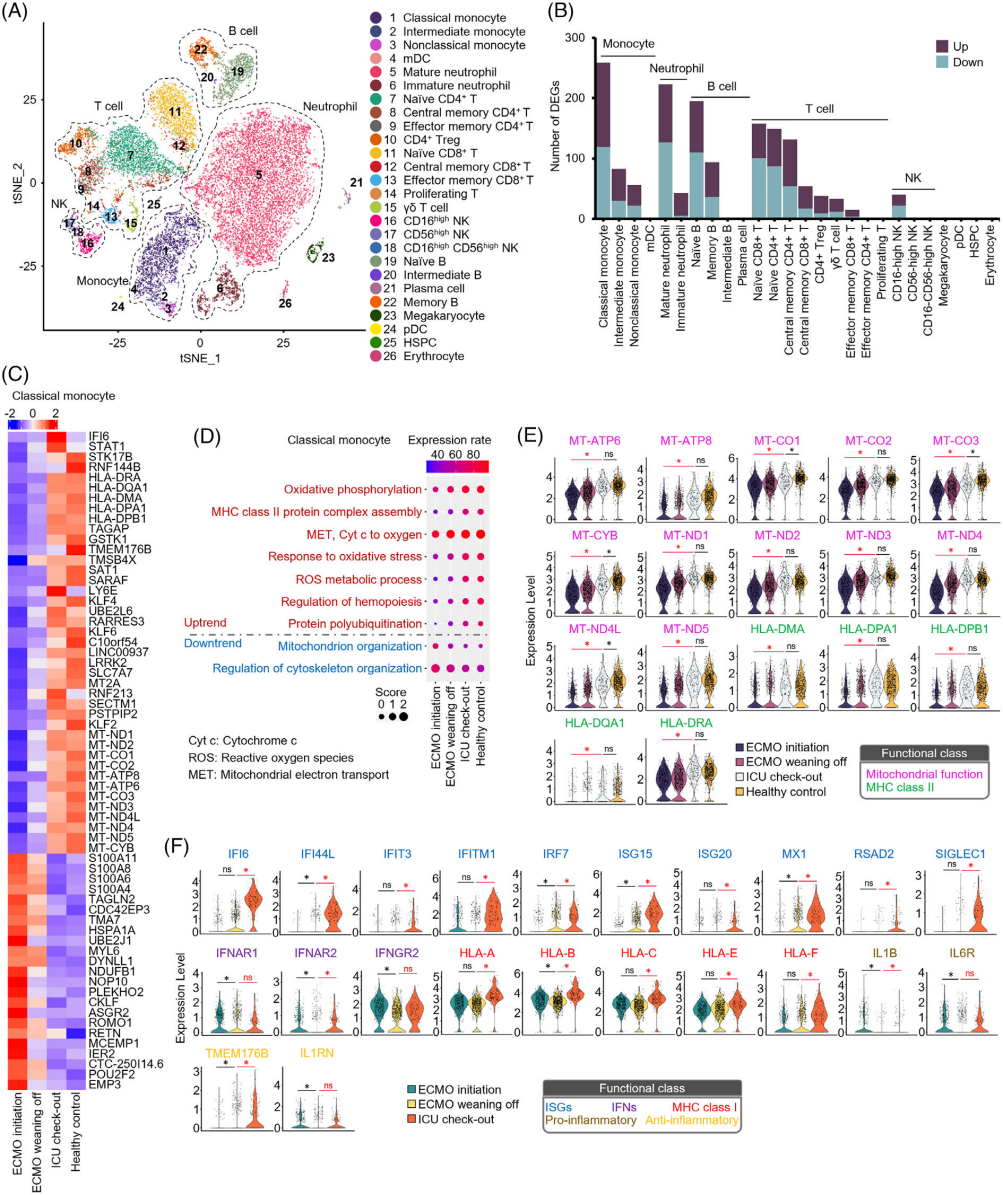
Transcriptomic characteristics of peripheral classical monocytes. (A) T‐distributed stochastic neighbour embedding plots of a total of 22 383 peripheral blood cells from acute respiratory distress syndrome and healthy participants (extracorporeal membrane oxygenation [ECMO] initiation: 2743; ECMO weaning: 4124; intensive care unit (ICU) checkout: 9121; healthy controls: 6395). Each colour represents a cell type. (B) The number of differentially expressed genes of each subset from healthy controls and the point of ECMO initiation. (C) Heatmap of genes screened out from classical monocytes of ECMO initiation, ECMO weaning off, ICU checkout and healthy control. (D) Gene ontology (GO) analysis of screened genes in C (chi‐squared test, *p* < 0.05). The colour of the dots from blue to red indicates the low to high expression rate of genes that were included in the corresponding GO term in each sample. Dot size is relative to the score of the GO term in each sample. (E) Violin plots of selected genes that were involved in mitochondrial function and major histocompatibility complex class II from ECMO initiation, ECMO weaning off, ICU checkout and healthy controls. (F) Violin plots of selected genes that were involved in interferon‐stimulated genes, interferon receptors, MHCI molecules, pro‐inflammatory and anti‐inflammatory factors from ECMO initiation, ECMO weaning off and ICU checkout.

A set of transcripts was screened in mature neutrophils that were positively or negatively associated with disease improvement (Figure 4A). The upregulated transcripts in this set were enriched in the biological process of mitochondrial functional improvement (Figure 4B,C). We found that apoptosis rates were significantly decreased after disease improvement, suggesting that the high expression of mitochondrial functional genes is not caused by excessive cellular apoptosis (Supplementary Figure 4). In contrast, the expression of genes necessary for the migration and adhesion, including FLNA, CD44, MYADM and AQP9, were downregulated (Figure 4C). Meanwhile, HCK, FGR and PECAM1, which are related to the phagocytosis, gradually returned to normal levels after ECMO weaning (Figure 4C). The expression of ISG and MHCI‐related transcripts remained lower during ECMO but was upregulated at ICU checkout (Figure 4D).

**FIGURE 4:**
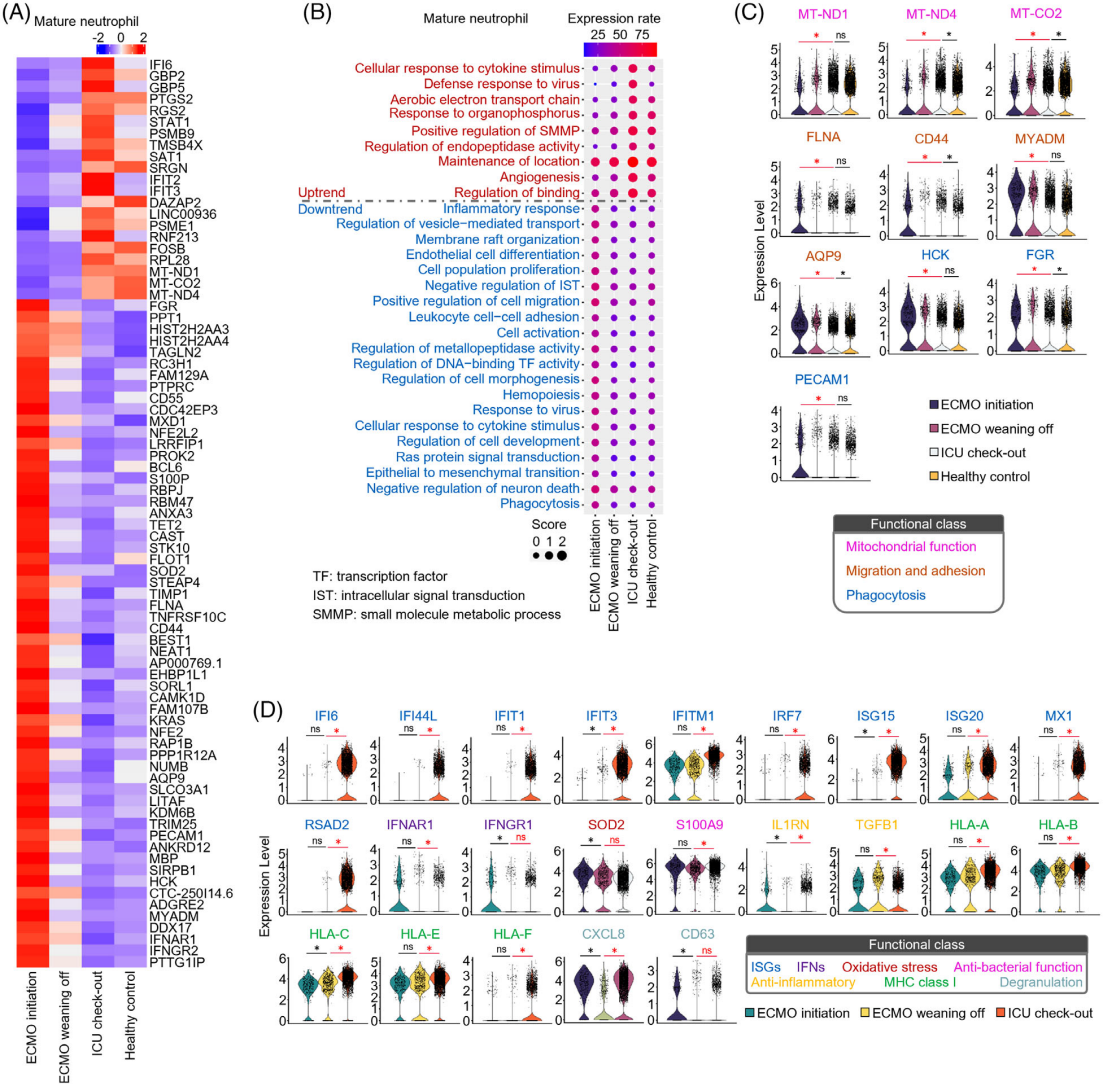
Dynamic transcriptomic characteristics of peripheral mature neutrophils. (A) Heatmap of genes screened out from mature neutrophils of extracorporeal membrane oxygenation (ECMO) initiation, ECMO weaning off, ICU checkout and healthy control. (B) Gene ontology (GO) analysis of screened genes in A (chi‐square test, *p*‐value < 0.05). The colour of the dot from blue to red indicates the low to high expression rate of genes that were included in the corresponding GO term in each sample. Dot size is relative to the score of the GO term in each sample. (C) Violin plots of selected genes that were involved in mitochondrial function, migration, adhesion, phagocytosis, metallopeptidase inhibitor and regulation of endothelial cell differentiation from ECMO initiation, ECMO weaning off, ICU checkout and healthy control. (D) Violin plots of selected genes that were involved in interferon‐stimulated genes, interferon receptors, oxidative stress, anti‐bacterial function, anti‐inflammatory, MHCI molecules and degranulation from ECMO initiation, ECMO weaning off and ICU checkout.

We also found megakaryocytes communicated with haematopoietic stem cells via transforming growth factor–beta signalling to reduce ECMO‐associated bleeding risk (Supplementary Figure 5).

As an invasive examination that rarely alters clinical treatment strategies, lung biopsy is not widely utilized in ARDS patients, which also makes it difficult to explore the cellular functionality and communication characteristics during the pulmonary injury repair phase. To the best of our knowledge, this is the first study to describe dynamic changes in peripheral blood cells and lung tissue at the single‐cell transcriptomic level in patients with PARDS with ECMO support.

This study had several limitations. First, substantial immunological maturation occurs between ages 1 and 10 years, which may limit the generalizability of our study findings to other age groups. Second, we cannot fully differentiate between the ARDS and ECMO effects on neutrophils and other immune cells. Third, we acknowledge that the ideal samples should be collected from individuals of the same sex and age group. However, due to the scarcity of samples, we can only use the best samples available from children as the control group. Although this was a quick glance, these data provided insight into the characteristics of the immunological microenvironment and lung tissue repair required for the recovery of children with ARDS.

## CONFLICT OF INTEREST STATEMENT

No disclosures were reported.

## Supporting information

Supporting Information

Supporting Information

Supporting Information

## Data Availability

Multi‐omics data are openly available in GEO bank (GSE223793) at https://www.ncbi.nlm.nih.gov/geo/. Clinical data are available from the corresponding authors upon reasonable request and with the permission of the institution.
