# Supplementary material for: Transcriptomic evidence of lung repair in paediatric ARDS survival
Source: Clin Transl Med. 2023 Aug 17;13(8):e1366. doi: 10.1002/ctm2.1366 (PMC10435684; doi:10.1002/ctm2.1366)
Supplement: Supplementary file 3 — Supporting Information [file CTM2-13-e1366-s002.docx]

**Supplementary Appendix**

**Materials and methods**

**Human subject.** The surviving patient described in this case study was a 10-year-old girl admitted to the Chinese People's Liberation Army (PLA) General Hospital with ARDS. During the surgical repair of an air leak, a lung biopsy was subjected to a pathological examination and single-cell RNA sequencing (scRNA-seq). Blood samples for scRNA-seq were collected at three time points: ECMO initiation, ECMO weaning off, and intensive care unit (ICU) check-out. A control blood sample was also collected from a 15-year-old girl with heart failure prior to heart transplantation. Additionally, control lung samples were collected from adjacent healthy lung tissue near the lesion from a 1-year-old boy with lung sequestration and a 3-year-old boy with metastatic hepatoblastoma. Written and verbal authorisations were obtained for the application and disclosure of the patient’s protected health information. Furthermore, the medical ethics committee of Chinese PLA General Hospital approved this study of all blood samples and lung tissues (no. 2022052701006). Assessments of radiologic and microbiological changes and laboratory monitoring were also performed in the clinical care setting.

**Sample collection and scRNA-seq.** From each participant, 5 mL of venous blood was collected in a BD Vacutainer EDTA Tube (cat. #367861). The blood samples were immediately incubated at 20°C for 20 min. After being diluted with Hank’s balanced saline solution (Solarbio, Beijing, China; cat. #H1025), the samples were subjected to density gradient centrifugation to obtain peripheral blood mononuclear cells (PBMCs) in Ficoll-Paque medium. The lung tissues were washed three times with 1× phosphate buffered saline (PBS) and minced to 0.2–0.6 mm^3^ using scissors to prepare the homogenate buffer. The homogenates were washed with 20 mL of 1× PBS. For each homogenate, a 40-μm strainer was used to collect the minced lung tissues. The washed small pieces of lung tissue were resuspended in 10 mL of enzyme mix solution containing 50 caseinolytic U/mL dispase (Corning Laboratory Products, Corning, NY, USA; cat. #354235), 2 mg/mL collagenase, 1 mg/mL elastase, and 30 μg/mL DNase. The resuspended lung homogenates were digested at 37°C for 1 h and shaken every 10 min. Enzymatic hydrolysis was inhibited using 20 mL of PBS containing 10% foetal calf serum (FCS). The digested homogenates were centrifuged at 300 × *g* for 10 min after being passed through a 70-μm strainer. The red blood cells were lysed with 5 mL of red blood lysis buffer for 2 min at room temperature. After the termination reaction using 20 mL of PBS with 10% FCS, the cell suspensions were centrifuged at 300 × *g* for 10 min and passed through a 70-μm strainer. The clear cells were resuspended in 1 mL of PBS at 0°C, with single-cell suspensions having concentrations of 7–12 × 10^5^ cells/mL. The cell viabilities of the single-cell suspensions of the lung tissues and PBMCs were 56–75% and >80%, respectively. Each single-cell suspension was used to construct a single-cell RNA library using a 5’ kit v2 (10× Genomics, Pleasanton, CA, USA; cat. #PN-1000263) according to the 10× Genomics® Cell Preparation Guide^1,2^.

**Single-cell RNA-seq data analysis.** The raw scRNA-seq data were aligned and counted against the human reference genome (GRCh38) using Cell Ranger (version 4.0.0) to obtain the gene–cell matrices. After the doublet cells were removed using DoubletFinder (version 2.0.3), the matrices were submitted to the Seurat package (version 4.1.1) for quality control, batch-effect removal, dimension reduction, and clustering analysis. For quality control, the samples with total RNA counts of 200–6,000 and mitochondrial gene percentages <10% were used. The top 2,000 most variable genes were obtained for dimensional reduction by the “FindVariableFeatures,” “ScaleData,” and “RunPCA” functions. After batch-effect removal using the Harmony package (version 0.1.0), the “FindNeighbors,” “FindClusters,” and “RunTSNE” functions were performed to identify the cell clusters. Finally, the two-dimensional profiles were visualised using t-distributed stochastic neighbour embedding. The cell types were identified based on the canonical marker genes^1^ (Supplementary Figures 2, 3).

**Gene expression and functional enrichment analysis.** The analysis and processing of the scRNA-seq data were performed using R statistical language^1^. Based on the Seurat package, the differentially expressed genes of each cell type having more than 20 cells were calculated and visualised by the “FindMarkers” and “VlnPlot” functions (Bonferroni corrected adjusted *p* < 0.05), respectively. Heatmaps of the gene expression levels were generated using Complex Heatmap software (version 2.12.0). Biological processes were enriched based on Gene Ontology (GO) analysis with values of *p* < 0.01 (cumulative hypergeometric distribution) using Metascape^3^ (version 3.5; <https://metascape.org/>). The GO terms were visualised and scored using the gene-set module in the Seurat package. The apoptosis scores were assessed with values of *p* < 0.05 (Student’s t test) using AddModuleScore based on HALLMARK APOPTOSIS (M5902; https://www.gsea-msigdb.org).

**Cellular communication analysis.** For the cellular communication analysis, the cell–cell communication of each subtype with >10 cells was analysed (*p* < 0.05 using a permutation test) and visualised using CellChat (version 1.4.0)^4^.

**Data sharing statement.** Multiomics data are openly available in GEO bank (GSE223793; https://www.ncbi.nlm.nih.gov/geo/). Clinical data are available from the corresponding authors upon reasonable request and with the permission of the institution.

**References**

1. Li, K., et al. DJ-1 governs airway progenitor cell/eosinophil interactions to promote allergic inflammation. J Allergy Clin Immunol 150, 1178-1193.e1113 (2022).

2. Wang, Z., et al. Single-cell RNA sequencing of peripheral blood mononuclear cells from acute Kawasaki disease patients. Nat Commun 12, 5444 (2021).

3. Zhou, Y., et al. Metascape provides a biologist-oriented resource for the analysis of systems-level datasets. Nat Commun 10, 1523 (2019).

4. Jin, S., et al. Inference and analysis of cell-cell communication using CellChat. Nat Commun 12, 1088 (2021).

**Supplementary figures**

**
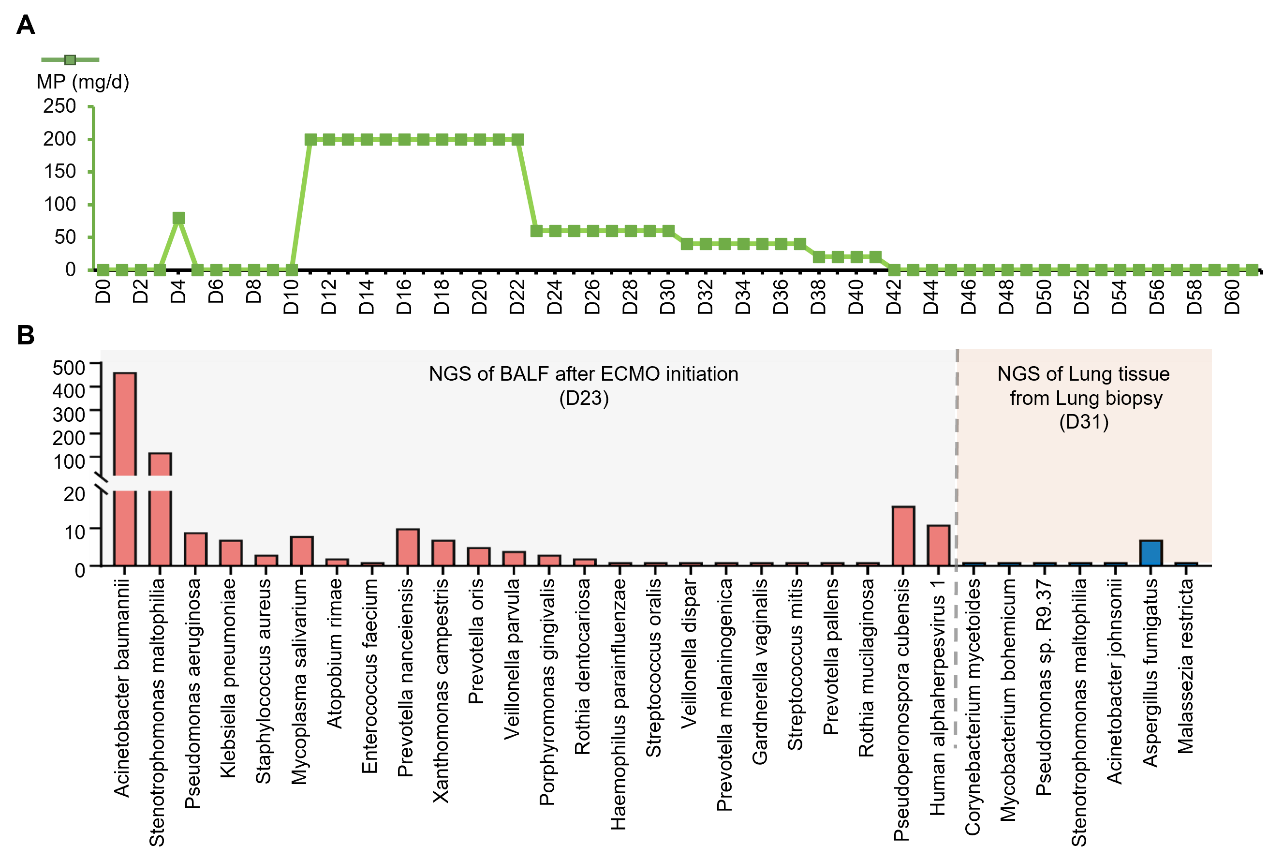
**

**Supplementary Figure 1. Application of methylprednisolone and metagenomic next-generation sequencing (Figure 1).** A. Application of methylprednisolone from mechanical ventilation to hospital discharge. B. Metagenomic next-generation sequencing of bronchoalveolar lavage fluid on day 23 (extracorporeal membrane oxygenation initiation) and lung tissue on day 31 (lung biopsy through video-assisted thoracoscopic surgery).


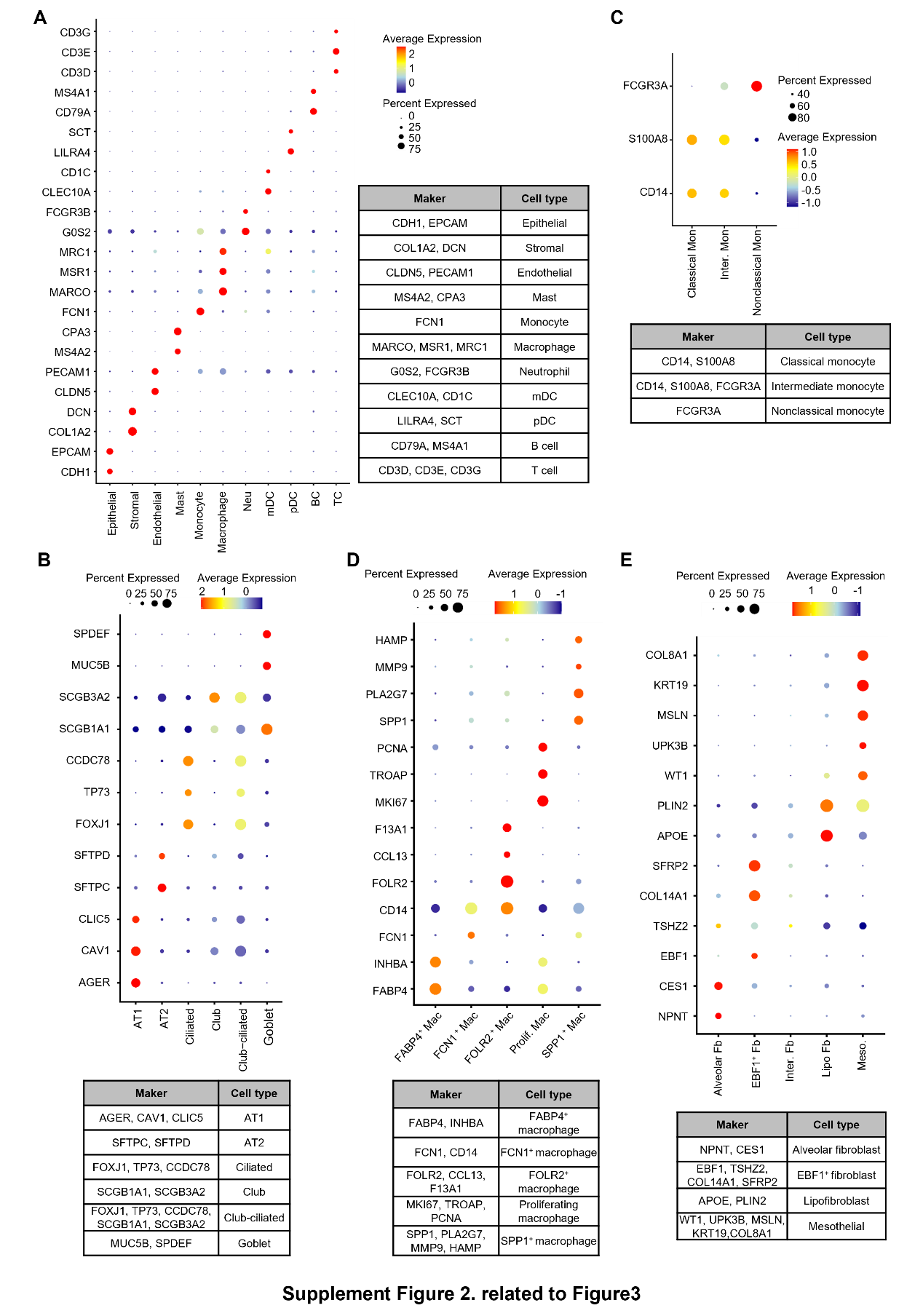


**Supplementary Figure 2. Identification of cell types in lungs (Figure 2).** A. Marker genes of each cell type. B–E. Marker genes of epithelial, monocyte, macrophage, and stromal subsets. The colour of the dots from blue to red indicates the low to high average expression level. Dot size is relative to the percentage of cells expressing genes.


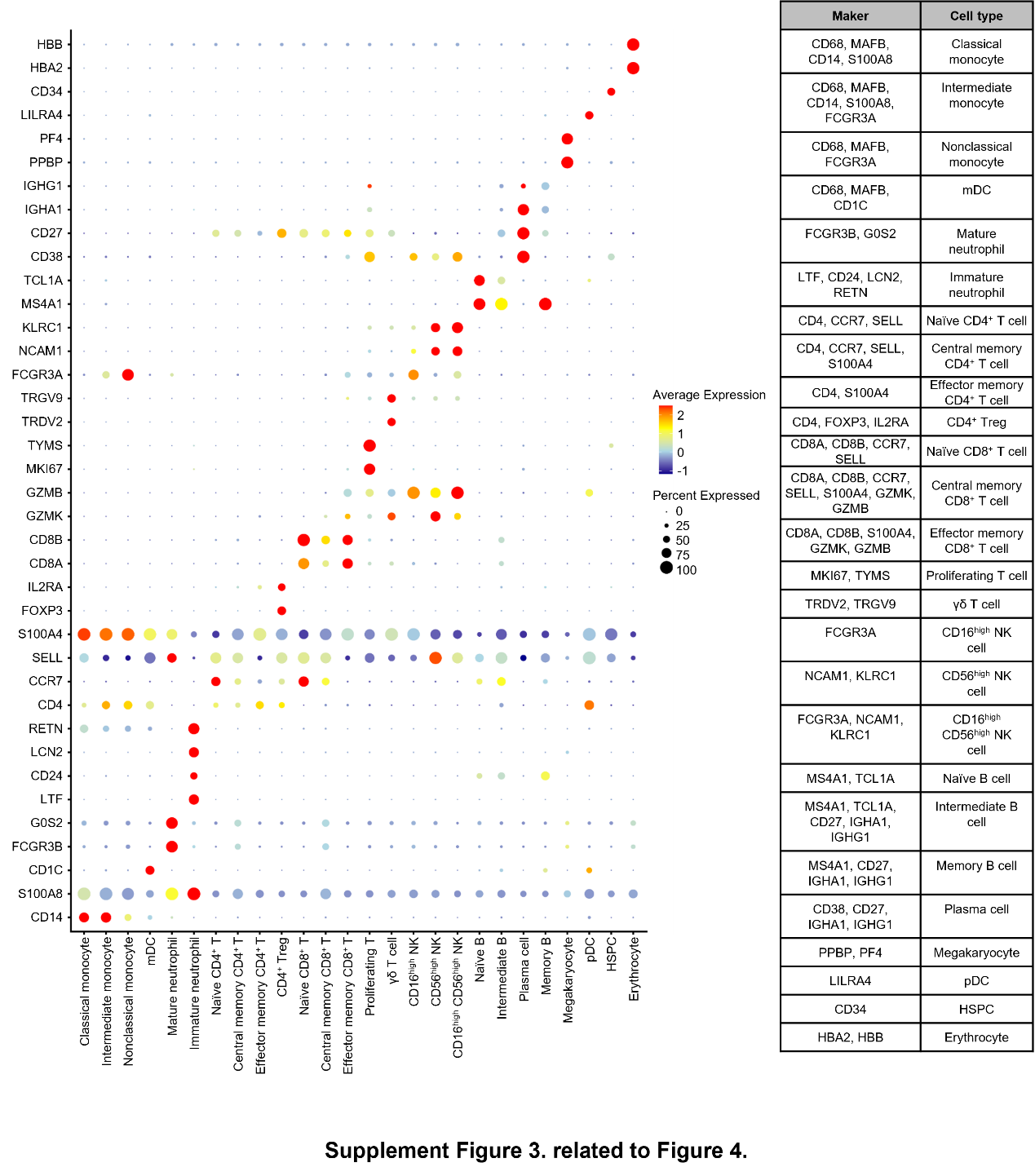


**Supplementary Figure 3. Identification of cell subsets in peripheral blood cells (Figures 3, 4).** Marker genes in each subset. The colour of the dots ranging from blue to red indicates the low to high average expression level. Dot size is relative to the percentage of cells expressing the genes.


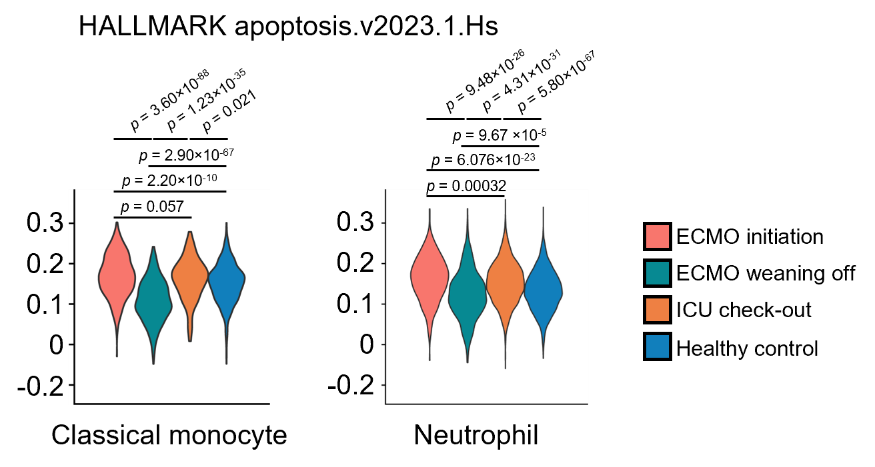


**Supplementary Figure 4.** Apoptosis scores for classical monocytes and mature neutrophils (*p* < 0.05, Student’s t test) (Figures 3, 4).


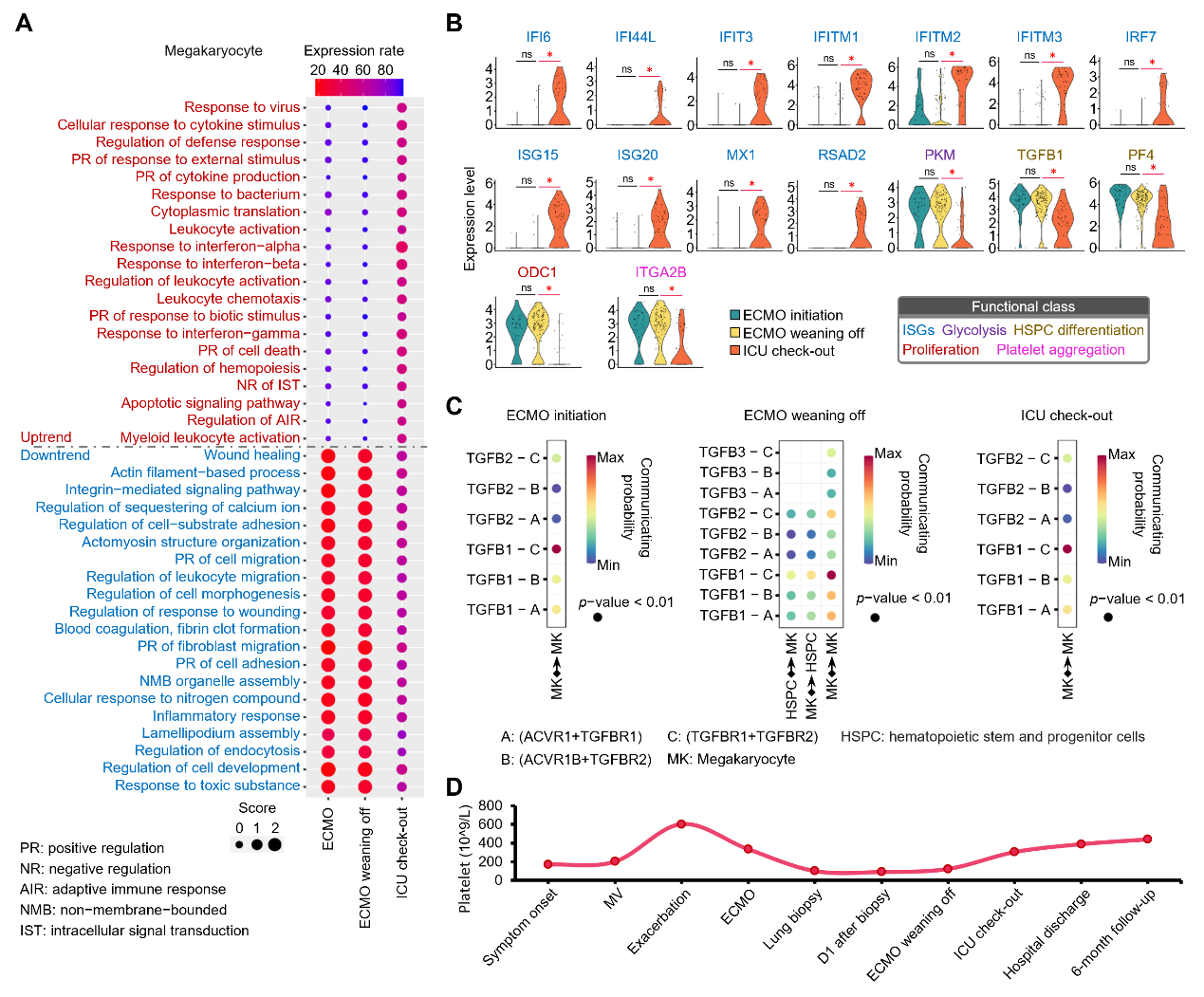


**Supplementary Figure 5. Enhanced communications between megakaryocytes and haematopoietic stem and progenitor cells (HSPCs).** A. Gene Ontology (GO) analysis of genes screened out from megakaryocytes from extracorporeal membrane oxygenation (ECMO) initiation, ECMO weaning off, and intensive care unit (ICU) check-out. B. Violin plots of selected genes that were involved in interferon-stimulated genes, glycolysis, HSC differentiation, proliferation, and platelet aggregation from ECMO initiation, ECMO weaning off, and ICU check-out. C Dot plot analysis showing megakaryocyte–HSPC interactions during ECMO initiation, ECMO weaning off, and ICU check-out. D. The quantity of blood platelets of the patient with acute respiratory distress syndrome at various time points throughout the clinical course.

| Supplementary Table 1. Pathogen detection with various methods during disease course | | | | |
| --- | --- | --- | --- | --- |
| Throat swab | Blood | Endotracheal aspirate (ETA) | Bronchoalveolar Lavage Fluid (BALF) | Sputum |
| SARS-CoV-2-RNA (-) (D1-23) |  |  |  |  |
| MP RNA (-) (D3) |  |  |  |  |
| Flu A RNA (-) (D4) | EBV-DNA (-) (D4) |  |  |  |
| Flu B RNA (-) (D4) | CMV-DNA (-) (D4) |  |  |  |
| RSV RNA (-) (D4) | EBV-CA-IgG (-) (D4) |  |  |  |
| PIV RNA (-) (D4) | EBV-CA-IgM (-) (D4) |  |  |  |
| AdV RNA (-) (D4) | EBV-EA-IgA (-) (D4) |  |  |  |
| MP RNA (-) (D4) | EBV-NA-IgG (-) (D4) |  |  |  |
| CP RNA (-) (D4) |  |  |  |  |
|  |  | CP-DNA (-) (D5) |  |  |
|  |  | Flu A RNA (-) (D5) |  |  |
|  |  | 09H1 RNA (-) (D5) |  |  |
|  |  | RSV RNA (-) (D5) |  |  |
|  |  | PIV RNA (-) (D5) |  |  |
|  |  | AdV DNA (-) (D5) |  |  |
|  |  | MP DNA (-) (D5) |  |  |
|  |  | HRV RNA (-) (D5) |  |  |
|  |  | Flu B RNA (-) (D5) |  |  |
|  |  | HCOV RNA (-) (D5) |  |  |
|  |  | H3N2 RNA (-) (D5) |  |  |
|  |  | Boca DNA (-) (D5) |  |  |
|  |  | HMPV RNA (-) (D5) |  |  |
|  | RSV-IgM (-) (D7) |  |  |  |
|  | AdV-IgM (-) (D7) |  |  |  |
|  | Flu A-IgM (-) (D7) |  |  |  |
|  | Flu B-IgM (-) (D7) |  |  |  |
|  | PIV-IgM (-) (D7) |  |  |  |
|  | MP-IgM (-) (D7) |  |  |  |
|  | CP-IgM (-) (D7) |  |  |  |
|  | LP-IgM (-) (D7) |  |  |  |
|  |  |  | CP-DNA (-) (D16) |  |
|  |  |  | Flu A RNA (-) (D16) |  |
|  |  |  | 09H1 RNA (-) (D16) |  |
|  |  |  | RSV RNA (-) (D16) |  |
|  |  |  | PIV RNA (-) (D16) |  |
|  |  |  | AdV DNA (-) (D16) |  |
|  |  |  | MP DNA (-) (D16) |  |
|  |  |  | HRV RNA (-) (D16) |  |
|  |  |  | Flu B RNA (-) (D16) |  |
|  |  |  | HCOV RNA (-) (D16) |  |
|  |  |  | H3N2 RNA (-) (D16) |  |
|  |  |  | Boca DNA (-) (D16) |  |
|  |  |  | HMPV RNA (-) (D16) |  |
|  | Culture:S. epidermidis (MRCNS) (D23) |  |  |  |
|  | Culture (-) (D24) | Smear staining: A. baumannii (CR-AB) (D24) |  |  |
|  |  |  | Culture:P. aeruginosa, serratia marcescens, A. baumannii (CR-AB) (D25) |  |
|  |  | Smear staining:A. baumannii (CR-AB) (D26) |  |  |
|  |  |  | Culture:A. baumannii (CR-AB), K. pneumoniae (D27) |  |
|  |  |  | Culture:P. aeruginosa (D28) |  |
|  |  |  | Culture:A. baumannii (CR-AB), K. pneumoniae (D29) |  |
|  | Culture (-) (D30) |  |  | Culture:A. baumannii (CR-AB) (D30) |
|  |  |  |  | Culture:A. baumannii (D31) |
|  |  |  | Culture:A. baumannii (CR-AB) (D32) |  |
|  | Culture (-) (D33) |  |  |  |
| RSV, respiratory syncytial virus; PIV, parainfluenza virus; AdV, adenovirus; MP, mycoplasma pneumoniae; CP, chlamydia pneumoniae; SARS-CoV-2, severe acute respiratory syndrome coronavirus 2; EBV, Epstein-Barr virus; CMV, cytomegalovirus; EBV-CA, EBV-capsid antigen; EBV-EA, EBV-early antigen; EBV-NA, EBV-nuclear antigen; LP, Legionella pneumophila; HRV, human rhinovirus; HCOV, human coronavirus disease; HMPV, human metapneumovirus; S. epidermidis, Staphylococcus epidermidis; MRCNS, methicillin-resistant Staphylococcus epidermidis; A. baumannii, Acinetobacter baumannii; CR-AB, carbapenem-resistant Acinetobacter baumannii; P. aeruginosa, Pseudomonas aeruginosa; K. pneumoniae, Klebsiella pneumoniae. | | | | |

Supplementary Table 2. Genes associated with lung repair in subpopulations of macrophage and fibroblast

| **Genes associated with lung repair in subpopulations of macrophage and fibroblast** | | | | | | | | | |
| --- | --- | --- | --- | --- | --- | --- | --- | --- | --- |
| **Symbol** | **Description** | **FABP4+ MAC (ARDS lung vs. Normal child lung (1Y))** | | | | **FABP4+ MAC (ARDS lung vs. Normal child lung (3Y))** | | | |
|  |  | avg_log2FC | p_val_adj | pct.1 | pct.2 | avg_log2FC | p_val_adj | pct.1 | pct.2 |
| IL10 | Interleukin 10 | 1.1200852 | 5.2461E-266 | 0.264 | 0.007 | 1.0632303 | 1.62906E-69 | 0.264 | 0.018 |
| AREG | Amphiregulin | ND | ND | ND | ND | 0.7152613 | 1 | 0.278 | 0.348 |
| VCAN | Versican | 2.8308234 | 5.7403E-291 | 0.688 | 0.225 | 2.8854265 | 7.2327E-185 | 0.688 | 0.158 |
| LGMN | Legumain | 1.7549884 | 4.11467E-37 | 0.531 | 0.505 | 1.584156 | 1.49606E-13 | 0.531 | 0.578 |
| CD163 | CD163 | 2.8354233 | 8.5666E-242 | 0.827 | 0.73 | 1.5007102 | 5.30681E-86 | 0.827 | 0.775 |
| SOD2 | Superoxide Dismutase 2 | 1.7447468 | 3.5156E-118 | 0.85 | 0.872 | 1.6135228 | 5.42441E-94 | 0.85 | 0.803 |
| TIMP1 | Tissue Inhibitor Of Metalloproteinases 1 | 4.2836554 | 0 | 0.963 | 0.887 | 4.5047136 | 0 | 0.963 | 0.767 |
| **Symbol** | **Description** | **FCN1+ MAC (ARDS lung vs. Normal child lung (1Y))** | | | | **FCN1+ MAC (ARDS lung vs. Normal child lung (3Y))** | | | |
|  |  | avg_log2FC | p_val_adj | pct.1 | pct.2 | avg_log2FC | p_val_adj | pct.1 | pct.2 |
| IL10 | Interleukin 10 | 1.261921 | 4.8406E-108 | 0.474 | 0.051 | 1.0862144 | 1.33548E-18 | 0.474 | 0.142 |
| AREG | Amphiregulin | -0.5717376 | 0.1048404 | 0.545 | 0.63 | -0.9251465 | 1 | 0.545 | 0.519 |
| VCAN | Versican | 1.2817906 | 1.9168E-98 | 0.912 | 0.747 | 1.2525964 | 6.67211E-28 | 0.912 | 0.691 |
| LGMN | Legumain | 0.4708921 | 1 | 0.524 | 0.579 | ND | ND | ND | ND |
| CD163 | CD163 | 3.1044436 | 0 | 0.981 | 0.477 | 1.7422342 | 2.67592E-80 | 0.981 | 0.777 |
| SOD2 | Superoxide Dismutase 2 | ND | ND | ND | ND | 0.3296 | 6.12052E-07 | 0.976 | 0.893 |
| TIMP1 | Tissue Inhibitor Of Metalloproteinases 1 | 3.8945302 | 0 | 0.999 | 0.927 | 4.3351515 | 5.2993E-122 | 0.999 | 0.828 |
| **Symbol** | **Description** | **FOLR2+ MAC (ARDS lung vs. Normal child lung (1Y))** | | | | **FOLR2+ MAC (ARDS lung vs. Normal child lung (3Y))** | | | |
|  |  | avg_log2FC | p_val_adj | pct.1 | pct.2 | avg_log2FC | p_val_adj | pct.1 | pct.2 |
| IL10 | Interleukin 10 | 0.8326773 | 1 | 0.46 | 0.297 | -0.5260009 | 1 | 0.349 | 0.444 |
| AREG | Amphiregulin | 1.1935889 | 1 | 0.619 | 0.312 | 1.0064939 | 1 | 0.619 | 0.222 |
| VCAN | Versican | 1.2579702 | 0.0322977 | 0.698 | 0.312 | 2.1448065 | 0.000610447 | 0.698 | 0.139 |
| LGMN | Legumain | -0.4218325 | 1 | 0.952 | 0.906 | -0.5770147 | 1 | 0.952 | 0.972 |
| CD163 | CD163 | 2.0443381 | 7.26354E-09 | 0.937 | 0.641 | -0.558658 | 1 | 0.127 | 0.5 |
| SOD2 | Superoxide Dismutase 2 | -0.5236637 | 1 | 0.825 | 0.812 | 0.4573691 | 1 | 0.825 | 0.861 |
| TIMP1 | Tissue Inhibitor Of Metalloproteinases 1 | 2.5019801 | 2.01543E-11 | 0.984 | 0.672 | 3.859093 | 1.33429E-10 | 0.984 | 0.611 |
| **Symbol** | **Description** | **SPP1+ MAC (ARDS lung vs. Normal child lung (1Y))** | | | | **SPP1+ MAC (ARDS lung vs. Normal child lung (3Y))** | | | |
|  |  | avg_log2FC | p_val_adj | pct.1 | pct.2 | avg_log2FC | p_val_adj | pct.1 | pct.2 |
| IL10 | Interleukin 10 | 1.2796836 | 7.5369E-19 | 0.528 | 0.081 | 1.1306717 | 1 | 0.528 | 0.212 |
| AREG | Amphiregulin | ND | ND | ND | ND | -0.7184305 | 1 | 0.482 | 0.333 |
| VCAN | Versican | 0.9263215 | 1.35685E-05 | 0.715 | 0.568 | 0.6395899 | 1 | 0.715 | 0.545 |
| LGMN | Legumain | 1.1877208 | 1.3011E-14 | 0.885 | 0.764 | 0.8816295 | 1 | 0.885 | 0.818 |
| CD163 | CD163 | 2.7498569 | 4.27202E-70 | 0.988 | 0.669 | 1.5048053 | 3.74265E-08 | 0.988 | 0.879 |
| SOD2 | Superoxide Dismutase 2 | ND | ND | ND | ND | ND | ND | ND | ND |
| TIMP1 | Tissue Inhibitor Of Metalloproteinases 1 | 3.1234679 | 1.57327E-66 | 0.997 | 0.878 | 3.2518275 | 1.37429E-14 | 0.997 | 0.818 |
| **Symbol** | **Description** | **EBF1+ Fb (ARDS lung vs. Normal child lung (1Y))** | | | | **EBF1+ Fb (ARDS lung vs. Normal child lung (3Y))** | | | |
|  |  | avg_log2FC | p_val_adj | pct.1 | pct.2 | avg_log2FC | p_val_adj | pct.1 | pct.2 |
| EGR3 | Early Growth Response 3 | 0.5703132 | 0.001298025 | 0.429 | 0.055 | 0.4917665 | 1 | 0.429 | 0.13 |
| TGFB1 | Transforming Growth Factor Beta 1 | 0.5920959 | 3.37598E-05 | 0.643 | 0.103 | 0.5144376 | 0.1739323 | 0.643 | 0.215 |
| TGFBI | Transforming Growth Factor Beta Induced | 1.4250575 | 1.37647E-09 | 0.75 | 0.103 | 1.6083637 | 1.18847E-18 | 0.75 | 0.081 |
| AREG | Amphiregulin | 1.1731487 | 1.50781E-06 | 0.536 | 0.062 | 1.5045397 | 2.74013E-22 | 0.536 | 0.018 |
| FGF7 | Fibroblast Growth Factor 7 | -0.6463053 | 1 | 0.536 | 0.377 | ND | ND | ND | ND |
| IL33 | Interleukin 33 | -0.572051 | 1 | 0.357 | 0.322 | -0.440976 | 1 | 0.357 | 0.444 |
| VEGFA | Vascular Endothelial Growth Factor A | -0.8872488 | 1 | 0.714 | 0.534 | 0.5812531 | 1 | 0.714 | 0.292 |
| PDGFRA | Platelet Derived Growth Factor Receptor Alpha | -0.5269068 | 1 | 0.75 | 0.479 | -0.6894185 | 1 | 0.75 | 0.799 |
| VCAN | Versican | ND | ND | ND | ND | 0.4911094 | 1 | 0.821 | 0.676 |
| CTGF | Connective Tissue Growth Factor | 0.3314605 | 1 | 0.714 | 0.295 | -0.9684945 | 1 | 0.714 | 0.528 |
| TIMP1 | Tissue Inhibitor Of Metalloproteinases 1 | 3.6136159 | 3.85107E-12 | 1 | 0.89 | 3.8982227 | 7.54231E-14 | 1 | 0.965 |
| **Symbol** | **Description** | **Lipo. Fb (ARDS lung vs. Normal child lung (1Y))** | | | | **Lipo. Fb (ARDS lung vs. Normal child lung (3Y))** | | | |
|  |  | avg_log2FC | p_val_adj | pct.1 | pct.2 | avg_log2FC | p_val_adj | pct.1 | pct.2 |
| EGR3 | Early Growth Response 3 | 0.7525697 | 0.2013851 | 0.518 | 0.114 | 0.7836931 | 1 | 0.518 | 0.12 |
| TGFB1 | Transforming Growth Factor Beta 1 | -0.4287628 | 1 | 0.85 | 0.914 | -0.9346489 | 1 | 0.85 | 0.72 |
| TGFBI | Transforming Growth Factor Beta Induced | 0.7900533 | 1 | 0.757 | 0.8 | 1.044243 | 1 | 0.757 | 0.44 |
| AREG | Amphiregulin | 0.5421268 | 1 | 0.479 | 0.457 | ND | ND | ND | ND |
| FGF7 | Fibroblast Growth Factor 7 | 0.7713694 | 1 | 0.704 | 0.457 | 1.0847528 | 0.008840443 | 0.704 | 0.16 |
| IL33 | Interleukin 33 | 1.3556409 | 0.1578839 | 0.607 | 0.257 | 1.2427658 | 1 | 0.607 | 0.24 |
| VEGFA | Vascular Endothelial Growth Factor A | 0.428825 | 1 | 0.786 | 0.4 | 0.4288901 | 1 | 0.786 | 0.44 |
| PDGFRA | Platelet Derived Growth Factor Receptor Alpha | 1.4242168 | 1.64416E-08 | 0.9 | 0.4 | 1.2995214 | 0.000113566 | 0.9 | 0.4 |
| VCAN | Versican | 0.5030423 | 1 | 0.736 | 0.571 | 0.480137 | 1 | 0.736 | 0.4 |
| CTGF | Connective Tissue Growth Factor | 1.8584161 | 2.33213E-06 | 0.736 | 0.143 | 1.0675241 | 1 | 0.736 | 0.24 |
| TIMP1 | Tissue Inhibitor Of Metalloproteinases 1 | 5.0716739 | 1.46379E-17 | 1 | 0.914 | 4.1273482 | 1.77194E-11 | 1 | 0.8 |
| MAC, macrophage; ARDS, acute respiratory distress syndrome; Lipo. Fb, lipofibroblast; avg_log2FC, average log2 fold change; p_val_adj, p-value adjusted; ND, non-detectable; vs., versus; pct.1, the percentage of cells where the gene is detected in the first group; pct.2, the percentage of cells where the gene is detected in the second group. | | | | | | | | | |
